# Protease-sensitive prions with 144-bp insertion mutations

**DOI:** 10.18632/aging.100543

**Published:** 2013-02-28

**Authors:** Xiangzhu Xiao, Ignazio Cali, Zhiqian Dong, Gianfranco Puoti, Jue Yuan, Liuting Qing, Heming Wang, Qingzhong Kong, Pierluigi Gambetti, Wen-Quan Zou

**Affiliations:** ^1^ Department of Pathology, Case Western Reserve University, Cleveland, OH 44106, USA; ^2^ Department of Neurology, Case Western Reserve University, Cleveland, OH 44106, USA; ^3^ National Prion Disease Pathology Surveillance Center, Case Western Reserve University, Cleveland, OH 44106, USA; ^4^ National Center for Regenerative Medicine, Case Western Reserve University, Cleveland, OH 44106, USA; ^5^ Department of Epidemiology & Biostatistics, Case Western Reserve University, Cleveland, OH 44106, USA; ^6^ State Key Laboratory for Infectious Disease Prevention and Control, National Institute for Viral Disease Control and Prevention, Chinese Center for Disease Control and Prevention, Beijing, China; ^7^ First Affiliated Hospital, Nanchang University, Nanchang, Jiangxi Province, China

**Keywords:** Prions, protease-sensitive prions, prion disease, insertion mutation, neuropathology, Western blotting, phenotype

## Abstract

Insertion of 144-base pair (bp) containing six extra octapeptide repeats between residues 51 and 91 of prion protein (PrP) gene is associated with inherited prion diseases. Most cases linked to this insertion examined by Western blotting showed detectable proteinase K-resistant PrP^Sc^ (rPrP^Sc^) resembling PrP^Sc^ type 1 and type 2 in sporadic Creutzfeldt-Jakob disease (sCJD), or PrP7-8 in Gerstmann-Sträussler-Scheinker disease. However, cases lacking detectable rPrP^Sc^ also have been reported. Which PrP conformer is associated with neuropathological changes in the cases without detectable rPrP^Sc^ remains to be determined. Here we report that while all six but one subjects with the 144-bp insertion mutations examined display the pathognomonic PrP patches in the cerebellum, one of them exhibits no detectable typical rPrP^Sc^ even in PrP^Sc^-enriched preparations. Instead, a large amount of abnormal PrP is captured from this case by gene 5 protein and sodium phosphotungstate, reagents that have been proved to specifically capture abnormal PrP. All captured abnormal PrP from the cerebellum and other brain regions is virtually sensitive to PK-digestion (termed sPrP^Sc^). The presence of the predominant sPrP^Sc^ but absence of rPrP^Sc^ in this 144-bp insertion-linked inherited CJD case suggests that mutant sPrP^Sc^ is the main component of the PrP deposit patches and sPrP^Sc^ is sufficient to cause neurotoxicity and prion disease.

## INTRODUCTION

Mutations of prion protein gene (*PRNP*) are associated with a group of inherited prion diseases that are characterized clinically by dementia, ataxia, and myoclonus and pathologically by spongiform de-generation, astrocytic gliosis, and neuronal loss [1]. Like sporadic and acquired forms of prion diseases, the molecular hallmark of inherited prion diseases is the deposition in the central nervous system (CNS) of an abnormal isoform of prion protein (PrP^Sc^) that is derived from a host-encoded cellular prion protein (PrP^C^) via a structural transition from α-helices into β-sheet structures [2]. However, unlike the other two forms of the diseases, the conversion of PrP^C^ into the pathological PrP^Sc^ in inherited prion diseases is believed to be spontaneously triggered by the mutated PrP allele (PrP^M^). The wild-type PrP allele (PrP^Wt^) may or may not be recruited into PrP^Sc^ by the PrP^M^ allele [3-10]. Regardless of distinct etiologies, the PrP^Sc^ molecules present in all human prion diseases share some common structural, physicochemical, and biological properties, including a β-sheet-rich structure, resistance to proteinase K (PK) digestion, insolubility in non-denaturing detergents, and infectivity [2]. It has been well-documented that the co-existence of PrP^C^ and PK-resistant PrP^Sc^ (rPrP^Sc^) is a prerequisite for the pathogenesis of various prion diseases; however, what type of PrP^Sc^ conformers are directly responsible for the PrP deposition in the brain and the neuropathological changes in the prion diseases remains poorly understood [11].

Phenotypes of inherited prion diseases are mainly determined by specific mutations and a polymorphism at codon 129 (methionine (M) and valine (V)) of *PRNP* [12]. Most point mutations of *PRNP* are associated with inherited conditions exhibiting phenotypes similar to the well-characterized sporadic Creutzfeldt-Jakob disease (sCJD) with a fairly rapid course, deposition of a typical rPrP^Sc^ (designated PrP27-30 including PrP^Sc^ type 1 or type 2) in CNS, and widespread of spongiform degeneration. However, a few point mutations and a non-sense mutation are linked to the phenotype of Gerstmann-Sträussler-Scheinker disease (GSS), characterized by a relatively chronic clinical course and the presence of intense amyloid plaques composed of unique rPrP^Sc^ fragments (designated PrP7-8) in the affected brains. A third set of inherited prion diseases are associated with insertions of one to nine octapeptide repeats, except for three that has never been reported [13-18]. These mutations are located in a nonapeptide (R1) and four octapeptides (R2 to R4) of the form P(H/Q)GGG(-/G)WGQ between residues 51 and 91 of PrP^Wt^ [19-20]. The phenotypic heterogeneity and allelic origin of PrP^Sc^ linked to insertion of 6 octapeptide repeats of PrP have been extensively characterized [21-25, 5, 26-29]. Although deposition of PrP in the cerebellum of affected brains is strikingly consistent, phenotypes, neuropathological changes, levels of rPrP^Sc^ and transmissibility between individuals are variable, at least the electrophoretic pattern of PrP. PrP species in the insertion cases with undetectable rPrP^Sc^ by the conventional assay [24] have not been further characterized. Addressing these important issues may shed light on the correlation between highly variable neuropathological changes and the chameleon-like conformations of PrP^Sc^.

Here we examined brain PrP and neuropathological changes in six cases carrying the 144-bp PrP insertion mutation. Neuropathologically, these cases exhibited spongiform degeneration, astrocytosis and multicore plaques with or without neuronal loss although the severity of these changes differed between cases or between areas of the same brain. However, all but one consistently had the deposits of PrP patches orientated perpendicular to the pial surface in the molecular layer of the cerebellum. Surprisingly, one of the cases displaying the cerebellar PrP patches revealed virtually no brain rPrP^Sc^ that was well represented in the other five cases. In contrast, this variant case was associated with a large amount of PrP species that was PK-sensitive but was captured by reagents including gene 5 protein (g5p) and sodium phosphotungstate (NaPTA), proven to specifically bind to insoluble and aggregated PrP regardless of its PK resistance [30-33].

## RESULTS

### Clinical information of the six subjects examined

Sixcases with 144-bp insertion mutation (fCJD^Ins^) were collected between 2000 and 2006 at the NPDPSC (Table 1). All six cases were female with average age at onset of 38.5 ± 9.8 years and highly variable disease durations ranging between 3 and 180 months. Two cases were methionine/methionine (M/M) homozygous at residue 129 of PrP, three valine/valine (V/V) homozygous and one M/V heterozygous. The insertion mutant allele was coupled with the 129M in the M/V heterozygous subject. The octarepeat region in the six fCJD^Ins^ cases examined included two types of sequences (Table 1).

**Table 1:** Clinical features of fCJDIns

Case #	Codon 129-mut. Allele	Insertion type^a^	PrP^Sc^ type	Age at onset (yrs)	Duration (months)	Sex	Symptoms at onset
1	VV-129V	1	1	51	6	F	Ataxia associated with cognitive decline.
2	VV-129V	2	1	33	180	F	Cognitive decline (mostly dyscalculia and visuospatial deficit).
3	MM-129M	2	2	26	144	F	Jerks and progressive cognitive decline characterized by apraxia and dyscalculia.
4	MV-129M	1	1	39	9	F	N/A
5	VV-129V	2	2	49	3	F	Headache and fatigue; weeks later, sleep disturbance and ataxia.
6	MM-129M	1	None^b^	33	60	F	Cognitive decline and headache.

Case #	Symptoms during the disease course						EEG^c^
	Parkinsonism	Ataxia	Myoclonus	Focal weakness	Psychosis	Seizure	
1	−^d^	+^e^	+	−	−	−	No typical, diffuse slowing
2	Not reported	not reported	not reported	−	+	−	No typical, diffuse slowing
3	−	−	+	−	−	−	N/A^f^
4	N/A	N/A	N/A	N/A	N/A	N/A	N/A
5	+	+	+	−	−	−	No typical, diffuse slowing
6	+	+	+	−	+	−	No typical, diffuse slowing

Case #	MRI	Family history of dementia
1	No typical signs of prion disease; brain atrophy particularly in the cerebellum and brainstem.	Mother: died of Alzheimer's disease at age 72.
2	No typical signs of prion disease; severe atrophy in the parietal occipital and cerebellar cortex.	No family history of neurodegenerative diseases.
3	N/A	High penetrance of autosomal dominant presenile dementia in the father's side.
4	N/A	N/A
5	No typical signs of prion disease.	Mother: died of corticobasal degeneration; mother's brother was diagnosed with Parkinson's disease.
6	No typical signs of prion disease; severe brain atrophy 4 years after disease onset.	Similar dementia in her father who became demented at age 42 and died at age 52.

a Two types of insertion mutations: 1: R1.R2.R2.R3g.R2.R2.R2.R2.R2.R3.R4; 2: R1.R2.R2.R3.R2.R2.R3g.R2.R2.R3.R4.

b No detectable rPrPSc.

c EEG: Electroencephalogram.

d Absent.

e Present.

f Not available.

### Detection of rPrP^Sc^ by conventional Western blot analysis

**Figure 1:**
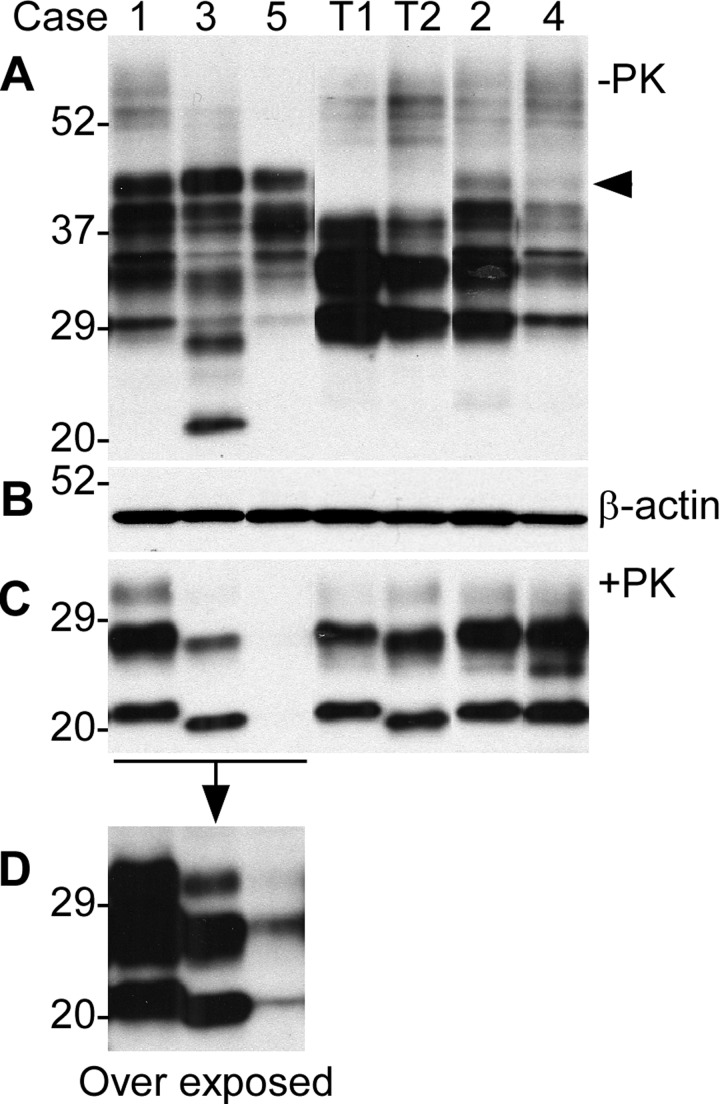
Detection of PrP in brains from five cases with six extra octapeptide repeats using Western blotting with 3F4. (**A**) Brain samples were from five cases (1 through 5) and were not treated with PK. T1: PrP^Sc^ type 1 control; T2: PrP^Sc^ type 2 control. (**B**) Western blot of β-actin, which was used to monitor the amounts of samples from each case. (**C**) The samples were treated with PK prior to SDS-PAGE and immunoblotting. The gel mobility of the PK-resistant PrP from the cases 1, 2, and 4 was similar to that of PrP^Sc^ type 1 control migrating at ~21 kDa while the case 3 was similar to PrP^Sc^ type 2 migrating at ~19 kDa. No PK-resistant PrP was visible in the case 5. (**D**) An over exposed smaller blot from the left part of the blot shown in **C**. The PK-resistant PrP bands from case 5 became detectable, the gel mobility of which was similar to that of case 2 migrating at ~19 kDa.

In the samples without PK-treatment, an extra band was observed migrating at ~38-40 kDa in the five cases with fCJD^Ins^ but not in PrP^Sc^ type 1 and type 2 controls from sporadic CJD (sCJD) (Fig. 1A, indicated by the arrow head). This extra high band represents the diglycosylated PrP^Ins^ with six extra octapeptide repeats and the monoglycosylated and non-glycosylated PrP species carrying the insertion were mixed with the three wild-type PrP bands, as indicated by the presence of multiple bands between 28 and 38 kDa compared to the relatively pure three bands in the samples from PrP^Sc^ type 1 and type 2 of sCJD (Fig. 1A). The bands below 29-30 kDa were endogenously N-terminally truncated PrP fragments such as in cases 2 and 3 (Fig. 1A). The amounts of samples loaded were monitored by the detection of β-actin (Fig. 1B). PK-resistant rPrP^Sc^ was detected in the brain homogenates from five out of six cases with fCJD^Ins^ by conventional Western blotting with 3F4, although in case 5 rPrP^Sc^ bands became visible only in the over-exposed film (Fig. 1C and 1D). The lower PrP band of rPrP^Sc^ had the gel mobility of 21 kDa (identical to that of PrP^Sc^ type 1) for cases 1, 2, and 4, or ~19 kDa (identical to that of PrP^Sc^ type 2) for cases 3 and 5 (Fig. 1B and 1C; Table 1). Case 6 exhibited no typical rPrP^Sc^ by the conventional Western blot analysis (Table 1), which was further characterized extensively by enrichment using g5p and NaPTA, ultracentrifugation-based sedimentation, and two-dimensional gel electrophoresis as described in detail below.

### Detection of PrP^Sc^ in the case with no typical rPrP^Sc^

**Figure 2:**
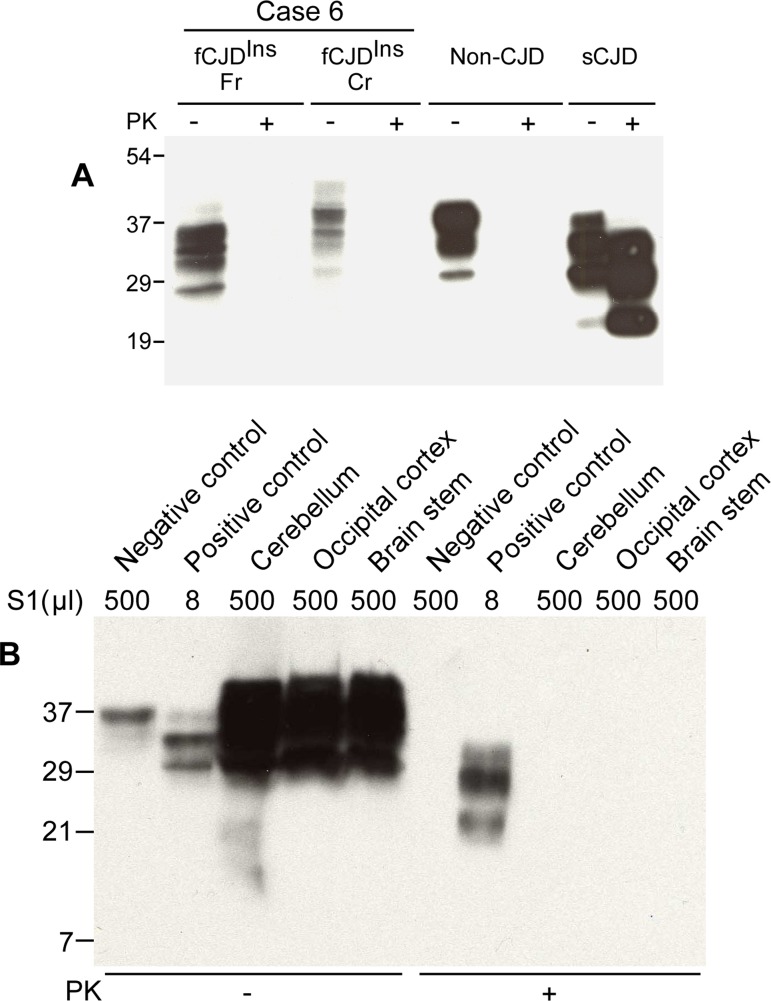
Detection of PK-sensitive PrP^Sc^. (**A**) Conventional Western blotting of PrP treated with or without PK in case 6. Fr: frontal cortex; Cr: cerebellum. No PrP was observed after PK treatment in the samples from both fCJD^Ins^ (case 6) and non-CJD. The PK-resistant PrP27-30 was indicated in the sample from sCJD. The migration of PrP from the cerebellum of case 6 was slightly slower than that of PrP from both non-CJD and sCJD controls. (**B**) Precipitation of abnormal PrP by NaPTA. S1 from non-CJD (500 μl), sCJD (8 μl), and case 6 (three brain regions: 500 μl each) was incubated with NaPTA and then was subjected to SDS-PAGE and immunoblotting with 3F4. Although a small amount of PrP was precipitated from non-CJD brain sample (500 μl of S1), no PK-resistant PrP fragments were detected. NaPTA was able to precipitate PrP from 8 μl of sCJD S1 (62.5-fold less than non-CJD S1) and the precipitated PrP was resistant to PK-digestion. Compared to non-CJD sample, NaPTA precipitated large amounts of PrP from three different brain regions of case 6 including the cerebellum (Cr), occipital cortex (Oc) and brain stem (BS). After PK-treatment of the NaPTA-precipitated PrP from case 6, no PrP bands were observed.

Case 6 was different from other five cases with the 144-bp insertion mutation (Fig. 1C) as no rPrP^Sc^ was detected by conventional Western blot in the brain homogenates from frontal cortex and cerebellum (Fig. 2A). Instead, there were multiple PrP bands prior to PK-treatment although the upper PrP band was also higher than that from non-CJD and sCJD controls (Fig. 2A). These observations were in agreement with the genetic finding that fCJD^Ins^ contains a PrP^Ins^ (Table 1). The PrP^Ins^ molecule may form additional set of three glycoforms including diglycosylated, mono-glycosylated, and unglycosylated PrP^Ins^. Hence, there are at least 6 PrP bands that are assumed to be detected by 3F4 antibody in the brain homogenate from fCJD^Ins^ including differently glycosylated PrP^Wt^ and PrP^Ins^. Nevertheless, it was surprising that no PK-resistant PrP fragments were detectable by the conventional analysis with 3F4 antibody (Fig. 2A). In addition, anti-C antibody also failed to detect PK-resistant PrP fragments (data not shown).

NaPTA is a reagent that has been demonstrated to specifically precipitate both sPrP^Sc^ and rPrP^Sc^ [31, 32, 34]. In order to increase sensitivity of detection by Western blotting and ELISA, NaPTA has been used to enrich small amounts of PrP^Sc^ in prion-infected peripheral organs where no PK-resistant PrP can be detected by the conventional assays [32, 35, 36]. To detect sPrP^Sc^ species and determine if there is a small amount of rPrP^Sc^ in this fCJD subject, a relatively large amount of brain homogenate was used to incubate with NaPTA. Compared to the normal control, a larger amount of PrP was precipitated by NaPTA (Fig. 2B). Surprisingly, the precipitated PrP was virtually completely digested by PK (Fig. 2B). No typical rPrP^Sc^ was detectable even in the over-exposed film (data not shown).

**Figure 3:**
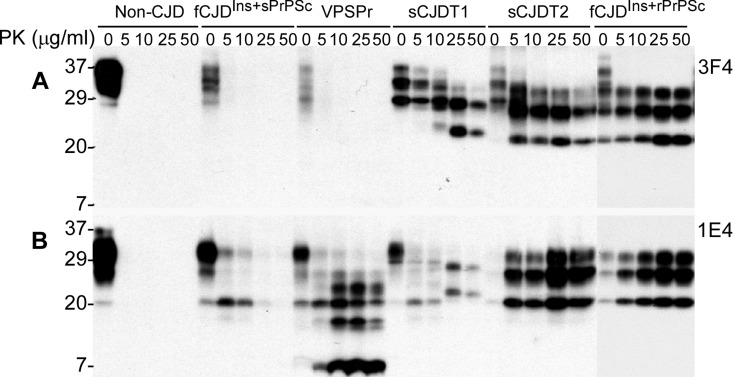
Determination of PK-resistant PrP^Sc^ with the 1E4 antibody. **A:** Brain homogenates from non-CJD, fCJD^Ins+sPrPSc^ (case 6), VPSPr with 129VV, sCJD type 1, sCJD type 2, and fCJD^Ins+rPrPSc^ were treated with a variety of concentrations of PK prior to SDS-PAGE and Western blotting with 3F4. PK-resistant PrP was only detected in sCJD type 1, type 2, or fCJD^Ins+rPrP^ but not in other cases. **B:** The PK-treated PrP from these cases were detected with 1E4. In contrast, PK-resistant PrP was also detected in both fCJD^Ins+sPrPSc^ and VPSPr, in addition to sCJD type 1, type 2, and fCJD^Ins+rPrPSc^. However, the PK-resistant PrP in fCJD^Ins+sPrPSc^ was only detected when treated with lower amounts of PK less than 25 μg/ml.

Using the anti-PrP monoclonal antibody 1E4 that has an epitope N-terminally adjacent to the 3F4 epitope [37, 38], our recent study identified a novel PK-resistant PrP^Sc^ characterized by the presence of dominant PK-sensitive PrP^Sc^ in an atypical human prion disease termed variably protease-sensitive prionopathy (VPSPr) [34, 39]. Upon PK-treatment, virtually no rPrP^Sc^ was detected by conventional Western blotting with 3F4, whereas PrP with a five-step ladder-like gel profile was detected preferentially by 1E4 in VPSPr [34, 39]. On the 3F4 blots, similar to non-CJD and VPSPr, no rPrP^Sc^ was detectable in brain homogenates from this fCJD^Ins^, whereas PK-rPrP^Sc^ was detected in sCJD type 1 and type 2 cases as well as in case 3 of fCJD^Ins^ (Fig. 3A). In contrast, on the 1E4 blots, except for non-CJD, PK-resistant PrP^Sc^ was detectable in cases 6 and 3 of fCJD^Ins^, VPSPr, sCJD type 1 and type 2. Nevertheless, rPrP^Sc^ in case 6 was detectable in the samples treated at PK concentration equal to or less than 10 μg/ml and decreased significantly at PK equal to or greater than 25 μg/ml (Fig. 3B). The amount and gel profile of rPrP^Sc^ in case 3 was very similar to those of rPrP^Sc^ in sCJD type 2. As demonstrated in our previous study [34, 39], rPrP^Sc^ from VPSPr exhibited a five-step ladder-like gel profile (Fig. 3B). The rPrP^Sc^ in case 6 detected by 1E4 was more similar to that of sCJD type 1 at low PK concentrations. However, at high PK concentration, no rPrP^Sc^ was detected from case 6 while the profile of typical PrP^Sc^ type 1 appeared in the sCJD type 1 case (Fig. 3B). Therefore, although no typical rPrP^Sc^ was detected in both fCJD^Ins^ of case 6 and VPSPr cases, the gel profile of rPrP^Sc^ detected by 1E4 is clearly different from that detected in VPSPr, classic sCJD, and fCJD^Ins^ of case 3. Importantly, the amounts and PK-sensitivity of rPrP^Sc^ detected by 1E4 in this fCJD^Ins^ (case 6) were also significantly less than those in VPSPr, sCJD and typical fCJD^Ins^ (case 3).

### Comparison of PrP oligomeric state between the two types of fCJD cases with the 144-bp insertion mutations

**Figure 4:**
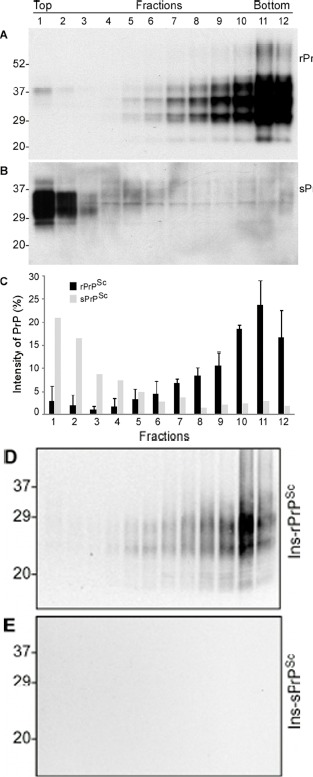
Comparison of oligomeric state of PrP in case 6 and other cases with rPrP^Sc^ by sucrose step gradient sedimentation. (**A**) Western blotting of PrP in individual fractions of sucrose gradient analysis of brain homogenate from case 3 with readily detectable rPrP^Sc^. (**B**) Western blotting of PrP in individual fractions of sucrose gradient analysis of brain homogenate from case 6 with sPrP^Sc^. **C:** Bar graph of PrP in individual fractions from three 144-bp insertion mutation cases with rPrP^Sc^ (average of PrP percentages from the three fCJD^Ins+rPrPSc^ cases) and case 6 with no rPrP^Sc^. Blots were probed with 3F4 antibodies. D and E: PrP in individual fractions from cases 3 (**D**) and 6 (**E**) was detected by Western blotting after treatment with PK at 0.5 μg/ml. PrP was only detected in fCJD^Ins+rPrPSc^ but not in fCJD^Ins+sPrPSc^.

To determine whether the PrP molecule in case 6 has a different oligomeric state compared to other fCJD^Ins^ cases, we further conducted the sedimentation of PrP^Sc^ in the sucrose step gradients. PrP in three cases with readily detectable rPrP^Sc^ was mostly recovered in bottom fractions 9-12, but not in top fractions (Fig. 4A and 4C), similar to classic sCJD [33]. In contrast, PrP in case 6 was mostly recovered in top fractions 1 and 2, while no significant increases in the amounts of PrP were detected in bottom fractions (Fig. 4B and 4C).

To investigate whether there are any rPrP^Sc^ species in different fractions in case 6, we treated PrP in the fractions with an extremely low concentration of PK. After treatment with PK at 0.5 μg/ml, no PrP was detected in all fractions from case 6 while PrP was detected in bottom fractions from case 3 (Fig. 4D and 4E). Our result suggested that PrP^Sc^ from fCJD^Ins^ case 3 was sensitive to PK even at 0.5 μg/ml.

### Detection of allelic compositions in PrP^C^ and PrP^Sc^ from fCJD^Ins^ with or without rPrP^Sc^ by sedimentation in detergents

**Figure 5:**
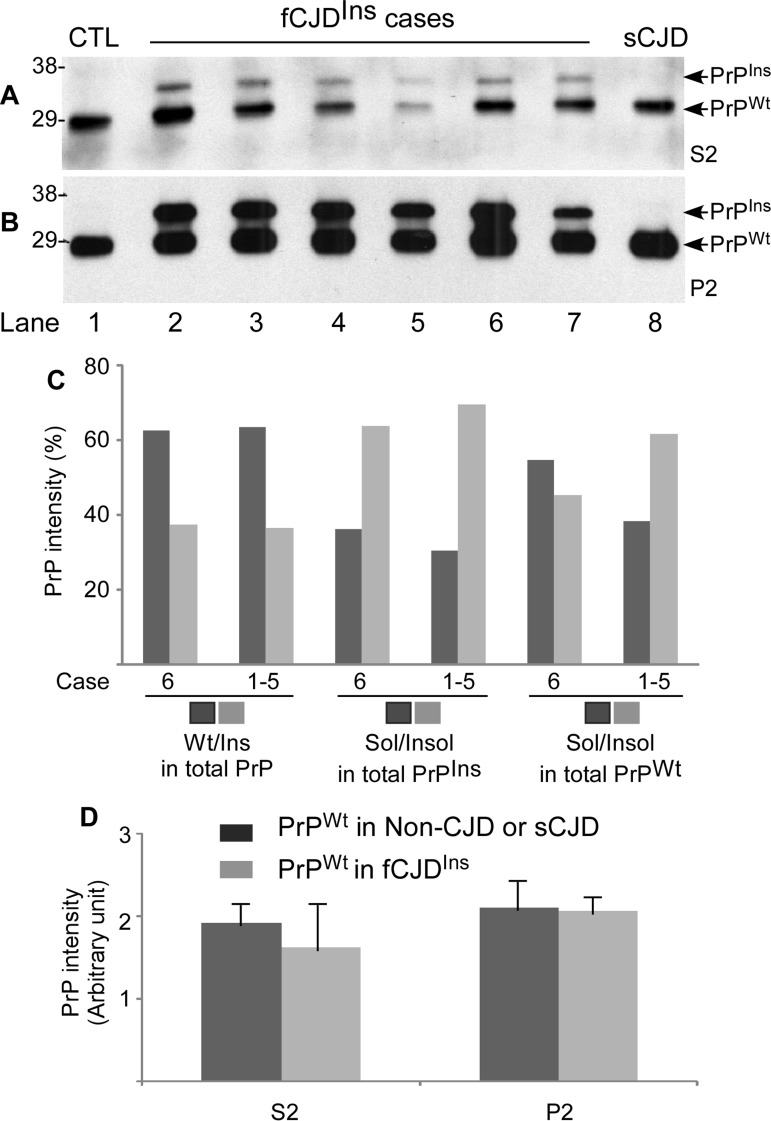
Determination of both PrP^Wt^ and PrP^Ins^ in detergent-soluble and -insoluble fractions by Western blotting. **A** and **B**: After ultracentrifugation in detergents, PrP in the detergent-soluble fraction (S2) (**A**) and -insoluble fraction (P2) (**B**) was detected by Western blotting with the anti-PrP antibody anti-N detecting the full-length PrP. Lane1: non-CJD control; Lane 2 fCJD^Ins+sPrPSc^ (case 6); Lanes 3 to 7: fCJD^Ins+rPrPSc^ (cases 1-5); Lane 8: sCJD control. **C**: Comparing compositions of total PrP, PrP^Ins^, and PrP^Wt^ in fCJD^Ins+sPrPSc^ (case 6) and fCJD^Ins+rPrPSc^ (cases 1-5, mean) by densitometric analysis of PrP intensity detected with Western blotting as shown in **A** and **B.** The left four bars exhibit the percentage of PrP^Wt^ or PrP^Ins^ in total PrP including soluble and insoluble forms. The central four bars exhibit the percentage of soluble or insoluble form in total PrP^Ins^. The right four bars exhibit the percentage of soluble or insoluble form in total PrP^Wt^. There were no significant differences in the percentage of PrP^Wt^ or PrP^Ins^ in total PrP between fCJD^Ins+sPrPSc^ and fCJD^Ins+rPrPSc^ (62.5/37.5 vs 63.4/36.6). Also there were no significant differences in the percentage of the soluble or insoluble form in PrP^Ins^ between the two conditions (36.2/63.8 vs 30.5/69.5). In contrast, the percentage of the soluble form of PrP^Wt^ was remarkably greater in fCJD^Ins+sPrPSc^ than in fCJD^Ins+rPrP^ (54.7% vs 38.4%), while the percentage of the insoluble form of PrP^Wt^ was significantly smaller in fCJD^Ins+sPrPSc^ than in fCJD^Ins+rPrPSc^ (45.3% vs 61.6%). **D:** Comparison of PrP^Wt^ intensity in S2 and P2 fractions between fCJD^Ins^ and non-CJD or sCJD. No differences in the levels of PrP^Wt^ from soluble or insoluble fraction were detected between fCJD^Ins^ and non-CJD or sCJD.

To dissect the differences in the composition of PrP^Wt^ and PrP^Ins^ between the two types of fCJD^Ins^ with rPrP^Sc^ or sPrP^Sc^ (fCJD^Ins+rPrPSc^ or fCJD^Ins+sPrPSc^), we analyzed the composition of PrP^Wt^ and PrP^Ins^. We took advantage of the most effective method by which the soluble PrP^C^ and insoluble PrP^Sc^ can be separated after ultracentrifugation in detergent buffer. After ultracentrifugation, PrP in the soluble and insoluble fractions was deglycosylated with PNGase F prior to detection by Western blotting with an antibody against N-terminal region of PrP called anti-N. In the samples from non-CJD and sCJD, only one PrP band was detected in supernatants (Fig. 5A, S2) and pellets (Fig. 5B, P2), migrating at ~28-29 kDa, which corresponds to the full-length deglycosylated PrP^Wt^. In contrast, in the samples from fCJD^Ins^, besides the ~28-29 kDa PrP^Wt^ band seen in non-CJD and sCJD samples, an additional PrP band migrating at ~33-34 kDa was detected in supernatants (Fig. 5A, S2) and pellets (Fig. 5B, P2), which corresponds to the full-length PrP^Ins^ with six octapeptide repeats containing 48 extra residues.

By densitometric analysis, we quantified distributions of PrP^Wt^ and PrP^Ins^ in S2 and P2 fractions in the two types fCJD^Ins+rPrPSc^ and fCJD^Ins+sPrPSc^. In both types of fCJD^Ins^, the ratio of PrP^Wt^ or PrP^Ins^ to the total PrP was the same: PrP^Wt^ accounted for 63%, whereas PrP^Ins^ accounted for 37% (Fig. 5C). Moreover, there were no differences in ratios of soluble and insoluble PrP^Ins^ to the total PrP^Ins^ between the two types of fCJD^Ins^ (Fig. 5C). In contrast, the ratio of soluble PrP^Wt^ to total PrP^Wt^ was significantly greater in fCJD^Ins+sPrPSc^ than in fCJD^Ins+rPrPSc^, whereas the ratio of insoluble PrP^Wt^ to total PrP^Wt^ was significantly smaller in fCJD^Ins+sPrPSc^ than in fCJD^Ins+rPrP^ (55% vs. 38% or 45% vs. 62%) (Fig. 5C).

Since fCJD^Ins^ contains only a single PrP^Wt^ allele whereas non-CJD or sCJD contains two PrP^Wt^ alleles, we assumed that the amount of PrP^Wt^ in fCJD^Ins^ should account for approximately half of total PrP^Wt^ detected in non-CJD or sCJD. To test for this possibility, we also quantified the intensity of the single PrP^Wt^ allele from fCJD^Ins^ and of total two PrP^Wt^ alleles from non-CJD and sCJD in both S2 and P2. Surprisingly, although fCJD^Ins^ contains only a single PrP^Wt^ allele, whereas non-CJD or sCJD contains two PrP^Wt^ alleles, the intensity of PrP^Wt^ detected in both S2 and P2 were similar between fCJD^Ins^ and non-CJD or sCJD (p > 0.05) (Fig. 5A, 5B and 5D).

### Detection of allelic compositions of PrP^C^ and PrP^Sc^ from the fCJD^Ins^ containing no rPrP^Sc^ by conformation-specific binding reagents

**Figure 6:**
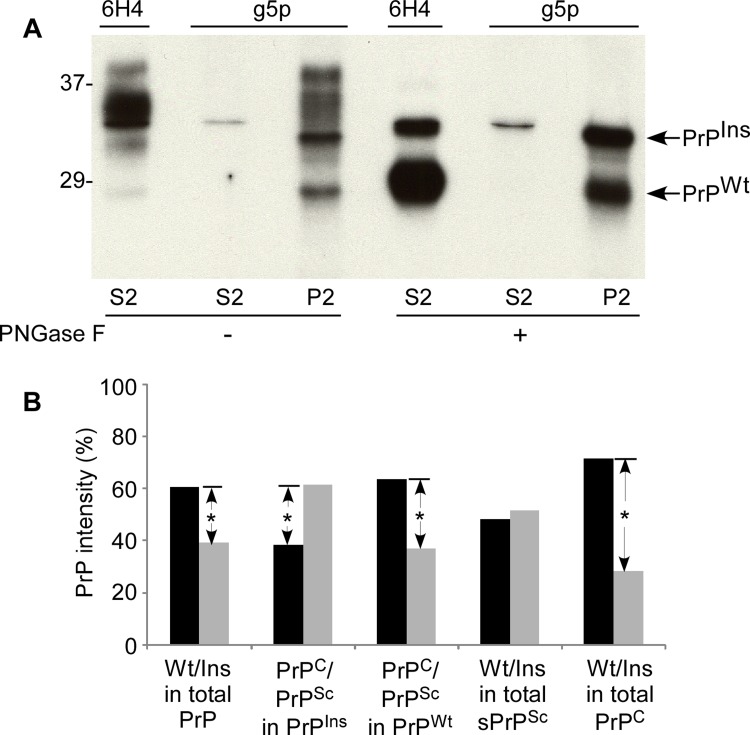
Allelic composition of PrP^Sc^ in fCJD^Ins+sPrPSc^. (**A**) Western blot analysis of PrP precipitated by either 6H4 or g5p. PrP from S2 or P2 was precipitated with 6H4 or g5p, respectively. The precipitated PrP was subjected to deglycosylation with PNGase F and followed by SDS-PAGE and immunoblotting with anti-N antibody. Although there are different profiles of PrP precipitated by 6H4 in S2 and by g5p in P2, they exhibit two bands in both preparations containing an upper band corresponding to full-length PrP^Ins^ and a lower band corresponding to full-length PrP^Wt^. (**B**) Quantitative analysis of allelic composition of PrP^Sc^ and PrP^C^ from either PrP^Ins^ or PrP^Wt^. The intensities of PrP treated with PNGase F on the blot shown in **A** were analyzed by densitometry. The PrP species precipitated from S2 by 6H4 was considered as PrP^C^ while PrP precipitated from S2 and P2 by g5p was considered as PrP^Sc^. Total PrP in fCJD^Ins+sPrPSc^ was composed of ~60% PrP^Wt^ and ~40% PrP^Ins^. Approximately 62% PrP^Ins^ was converted into PrP^Sc^ while ~38% remained as PrP^C^. In contrast, approximately 64% PrP^Wt^ remained as PrP^C^ while 36% was converted into PrP^Sc^. PrP^Sc^ was composed of 52% PrP^Ins^ and 48% PrP^Wt^ while PrP^C^ was composed of 28% PrP^Ins^ and 72% PrP^Wt^. These data represent averages from three independent experiments. *p < 0.05.

Taking advantage of anti-PrP antibody 6H4 and g5p that specifically recognizes either native PrP^C^ or misfolded PrP [40, 41, 30, 33], we dissected allelic composition of sPrP^Sc^ and ratio of sPrP^Sc^ to total PrP in the detergent-insoluble fraction (P2) rich in PrP^Sc^ from fCJD^Ins^. After specific capture of either PrP^C^ by 6H4 or PrP^Sc^ by g5p, the samples were treated with or without PNGase F that significantly decreases the heterogeneity of the protein by removal of the glycans (Fig. 6A).

Moreover, probing the captured proteins with anti-N antibody would simplify the recognition of the full-length PrP^Ins^ and PrP^Wt^. Without PNGase F treatment, several bands migrating between 28-39 kDa were detected in both 6H4-captured preparation from S2 preparation rich in soluble normal PrP and g5p-captured preparation from P2 preparation rich in insoluble abnormal PrP (Fig. 6A). These bands represent glycosylated and unglycosylated full-length PrP^Wt^ and PrP^Ins^. Notably, although g5p captured a large amount of misfolded PrP from P2 as expected, it also captured a small amount of PrP (a thin band) migrating at ~33-34 kDa that represents the unglycosylated full-length PrP^Ins^.

In the detergent-soluble fraction (S2) treated with PNGase F, two PrP bands were visualized by anti-N antibody in the preparation immunoprecipitated by 6H4: one with weaker intensity migrating at ~33-34 kDa corresponding to unglycosylated full-length PrP^Ins^ and another with stronger intensity migrating at ~28-29 kDa corresponding to unglycosylated full-length PrP^Wt^, while only one thin PrP band corresponding to unglycosylated full-length PrP^Ins^ was captured by g5p (Fig. 6A). In the P2 fraction rich in detergent-insoluble PrP, there were also two bands visualized by anti-N antibody in the sample precipitated by g5p. The gel mobility of the captured two bands was similar to that of the bands immunoprecipitated by 6H4 from S2 (Fig. 6A). Therefore, not only PrP^Ins^ but also PrP^Wt^ molecule participated in the formation of sPrP^Sc^ in the fCJD with the PrP insertion mutation.

Quantitative analysis by densitometry from three independent experiments revealed that PrP^Ins^ precipitated by 6H4 in S2 and by g5p in S2 and P2 accounted for ~40 % of total PrP while PrP^Wt^ precipitated by the two reagents accounted for ~60% of total PrP (Fig 6B), which was in agreement with those observed by the direct loading of soluble and insoluble PrP in Fig. 5A through C, suggesting that most PrP^Ins^ and PrP^Wt^ molecules in either normal or pathological isoforms were recovered by 6H4 and g5p. A detailed quantitative analysis was conducted in order to dissect the PrP composition of various PrP species (Fig. 6B). We observed that the majority of PrP^Ins^ (~62%) was converted into PrP^Sc^ while the minority (~38%) remained as PrP^C^ (Fig. 6B). In contrast, the majority of PrP^Wt^ (~64%) remained as PrP^C^ while ~36% of it was converted into PrP^Sc^. Also ~half of PrP^Sc^ was derived from PrP^Ins^ and another half from PrP^Wt^ (52% vs. 48%). However, PrP^C^ was composed of ~72% of PrP^Wt^ and ~28% of PrP^Ins^. Thus, these data were consistent with the results obtained in Fig. 5 and confirmed that the majority of PrP^Wt^ indeed was not converted into PrP^Sc^.

### Two-dimensional gel electrophoresis of PrP

To have a high resolution profile of PrP from fCJD^Ins^ containing both PrP^Wt^ and PrP^Ins^, the PrP molecule treated with or without PNGase F was subjected to the two-dimensional (2D) gel electrophoresis that is capable of separating proteins based on both molecular weight and charge.

**Figure 7:**
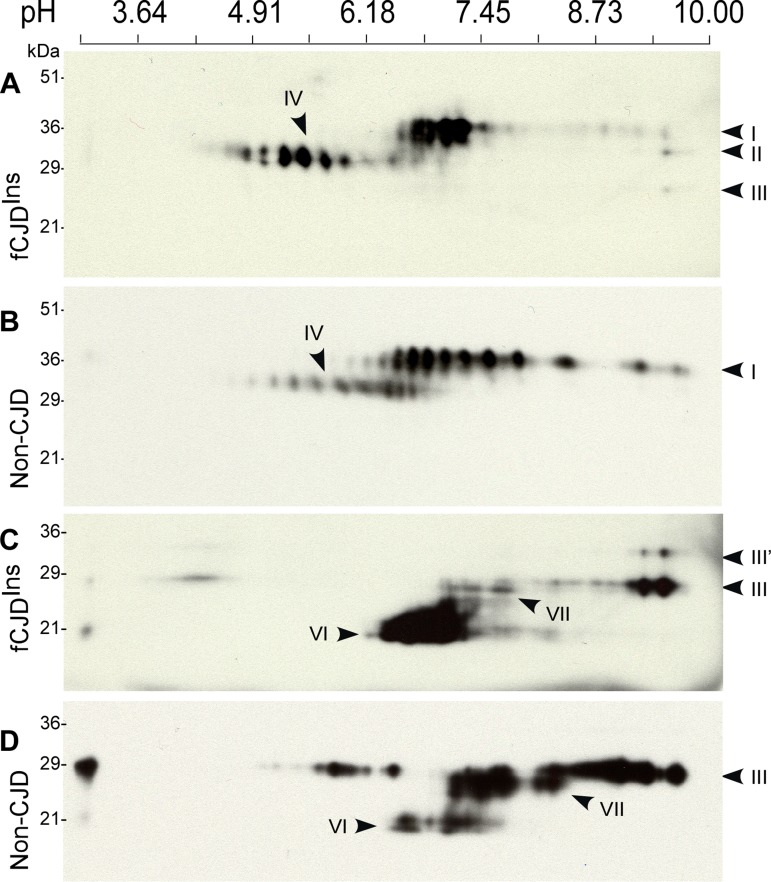
Two-dimensional Western blotting of PrP. (**A**) Comparison of untreated PrP from case 6 with fCJD^Ins+sPrPSc^ and non-CJD and sCJD. PrP from fCJD^Ins^ includes 2D spots I-IV and PrP from non-CJD mainly consists of 2D spots I and IV. (**B**) Comparison of PNGase F-treated PrP from fCJD^Ins^ and non-CJD. PrP from the two conditions comprises both full-length PrP (2D spots III) and N-terminally truncated PrP (2D spots VI); however, PrP from fCJD^Ins^ contains additional set of PrP spots (2D spots III’). The blots were probed with 3F4.

On 2D blots, PrP from non-CJD brain sample mainly was composed of two sets of PrP spots (Fig. 7B). The first set consisted of 13-14 PrP spots migrating between 33 and 42 kDa with *p*I 5.5-9.7, corresponding to diglycosylated, full-length PrP species (designated PrP 2D spots I). The second set comprised 9-10 PrP spots and was distributed between 29 and 32 kDa, *p*I 4.5-7.0, corresponding to diglycosylated, truncated PrP species (designated PrP 2D spots IV). This group of PrP species constituted the middle band of PrP on 1D blot.

The 2D profile of PrP from case 6 with fCJD^Ins^ was basically similar to that of PrP from non-CJD (Fig. 7A). However, several differences between the two were still detectable. For instance, *p*Is of the predominant PrP species from fCJD^Ins^ were different from those of non-CJD sample. Most PrP from 2D spots I of fCJD^Ins^ were mainly localized between *p*I 6.5 and 7.3 while intense PrP spots from that of non-CJD spread from *p*I 6.3-9.1. Compared to PrP from non-CJD, slight increases in the intensity of PrP 2D spots II, III, and IV were also observed (Fig. 7A). Moreover, the PrP 2D spots IV were more intense in fCJD^Ins^ than in non-CJD. These differences may result from the intrinsic nature of PrP in fCJD^Ins^, which is a mixture of PrP^Wt^ and PrP^Ins^.

Various PrP species from fCJD^Ins^ and non-CJD were also compared on 2D blot after deglycosylation that often profoundly decreases PrP heterogeneity. As expected, by probing with 3F4 antibody, two major sets of PrP spots were observed in the deglycosylated PrP from non-CJD: PrP 2D spots III migrating at 27-29 kDa with *p*I 7.0-9.6 corresponding to full-length PrP, and PrP 2D spots VI migrating at ~19-22 kDa with *p*I 6.1-8.1 corresponding to the N-terminally truncated PrP (Fig. 7D). However, in contrast to PrP from non-CJD, PrP from fCJD^Ins^ had an additional set migrating at 31-33 kDa with *p*I 9.0-9.5 designated PrP 2D spots III’ (Fig. 7C), which fitted well with the full-length PrP^Ins^ molecule with 6 extra repeats in terms of their molecular weight. In addition, we also observed that there was a new set of PrP spots designated PrP 2D spots VII present in the two conditions including fCJD^Ins^ and non-CJD, migrating at ~26-27 kDa with *p*I 5.0-8.1 (Fig. 7C and 7D). This group of PrP spots was N-terminally truncated PrP species missing ~10 residues from the farthest N-terminal portion. While PrP 2D spots III from the non-CJD brain sample were predominant, PrP 2D spots VI from fCJD^Ins^ were predominant. It is also intriguing to note that each of the PrP 2D spots III from the two conditions had different *p*I of the most intense PrP spot. For example, *p*I of the most intense PrP spot in fCJD^Ins^ was about 9.6, and ~9.0 in non-CJD. In view of their identical full-length PrP sequence, whether this difference is associated with distinct anchors remains to be further determined.

### Histopathology and immunohistochemistry

**Figure 8:**
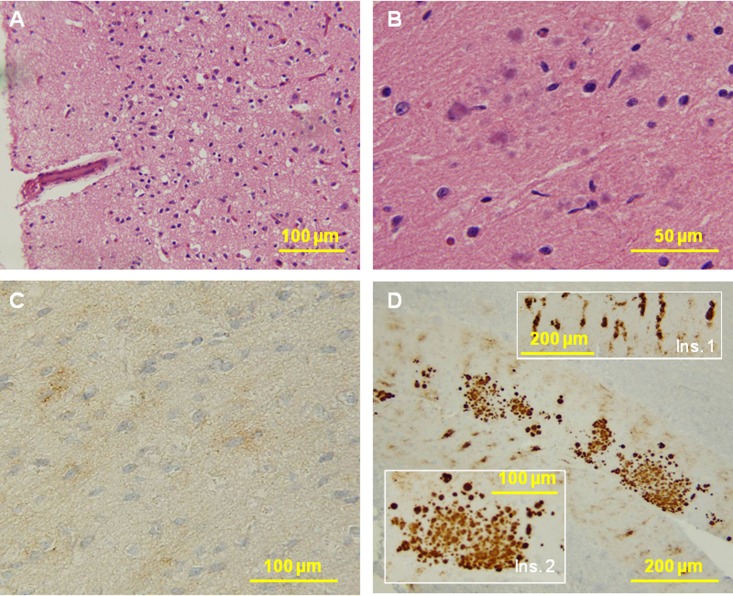
Histological and immunohistochemical examinations of brain tissues. **(A**) The cortex reveals no typical spongiform degeneration. (**B**) Several multicore plaques are present in the molecular layer of the cerebellum with a focal distribution. No pathology was seen in the pons and medulla. **C:** PrP immunostaining demonstrates weak staining with a synaptic pattern and occasional loose fine granular aggregates in the cerebral cortex. **D:** The molecular layer of the cerebellum shows a remarkable combination of strip-like staining (Ins. 1) and multicore plaques (Ins. 2). No immunostaining was seen in the pons and medulla.

The five fCJD^Ins^ cases associated with rPrP^Sc^ showed various degree of spongiform degeneration (SD) and variable astrogliosis and neuronal loss in the cerebral cortex and basal ganglia (Table 2 and Figure 8). Plaques with single or multiple cores were detected only in one case of the four cases with adequate number of slides in the molecular layer of the cerebellum. Typical kuru plaques were observed in the cerebellar granule cell layer and white matter.

**Table 2:** Neuropathological features of fCJDIns

Case #	Histopathology (H&E)			PrP immunostaining (IHC)			
	Cerebrum	Cerebellum		Cerebrum		Cerebellum	
	SD^a^	SD	Plaques	Synaptic with other pattern	Plaque/plaque-like	Plaque/plaque-like	Stripes
1	+	++	−	+	+	−	−
2	+++	NA	NA	+	+	NA	NA
3	+	++	−	+	+	−	++^b^
4	+	−	−	+	+	−	++^b^
5	+	−	+	+	+	+++/+^c^	+++
6	−	−	+	−	−	−/+++^c^	+++

a Spongiform degeneration;

b Not completely formed stripes;

c Granular layer + white matter/molecular layer. The severity of the SD and deposition of PrP IHC was scored as follow: absent (−), mild (+), moderate (++), and severe (+++).

PrP immunostaining showed the “synaptic” pattern in the cerebral cortex of all the cases of fCJD^Ins^ associated with rPrP^Sc^ (Table 2). However, the three cases VV-129 also showed the presence of prominent granules, micro plaques or plaque-like formations. Perineuronal staining was seen in the MV-129M and two VV-129V. Real plaques, often multicore, were seen in the 129-MM (case 6) and 129-VV (case 5) but not in the other cases.

Four of the five cases in which the cerebellum was available showed the distinctive stripe pattern in the molecular layer but the stripes of cases 3 and 4 of Table 2 were shorter or not completely formed. Stripes in the molecular layer could be either alone or associated with the synaptic or plaque-like formations in the granule cell layer and superficial white matter (VV-129, case 5). In the case lacking the stripe staining pattern, the staining of the molecular layer was synaptic (VV-129, case 1).

In fCJD^Ins^ case 6 lacking rPrP^Sc^, the histology of the brain areas examined revealed no SD (Fig. 8A). Several multicore plaques were present in the molecular layer of the cerebellum with focal distribution (Fig. 8B).

PrP immunostaining demonstrated weak staining with a focal synaptic pattern and occasional loose fine granular aggregates in the cerebral cortex (Fig. 8C). The molecular layer of the cerebellum had a remarkable combination of stripe-like staining so called PrP patches that are pathognostic for insertion mutation (Fig. 8D, ins. 1) and multicore plaques (Fig. 8D, ins. 2).

In conclusion, the case with sPrP^Sc^ (case 6) differed from those associated with rPrP^Sc^ by the lack of typical SD, and the presence of multicore plaques in limited regions of the cerebellar molecular layer. However had similar PrP stripes in the cerebellar molecular layer as the fCJD cases associated with rPrP^Sc^.

### Histoblotting

**Figure 9:**
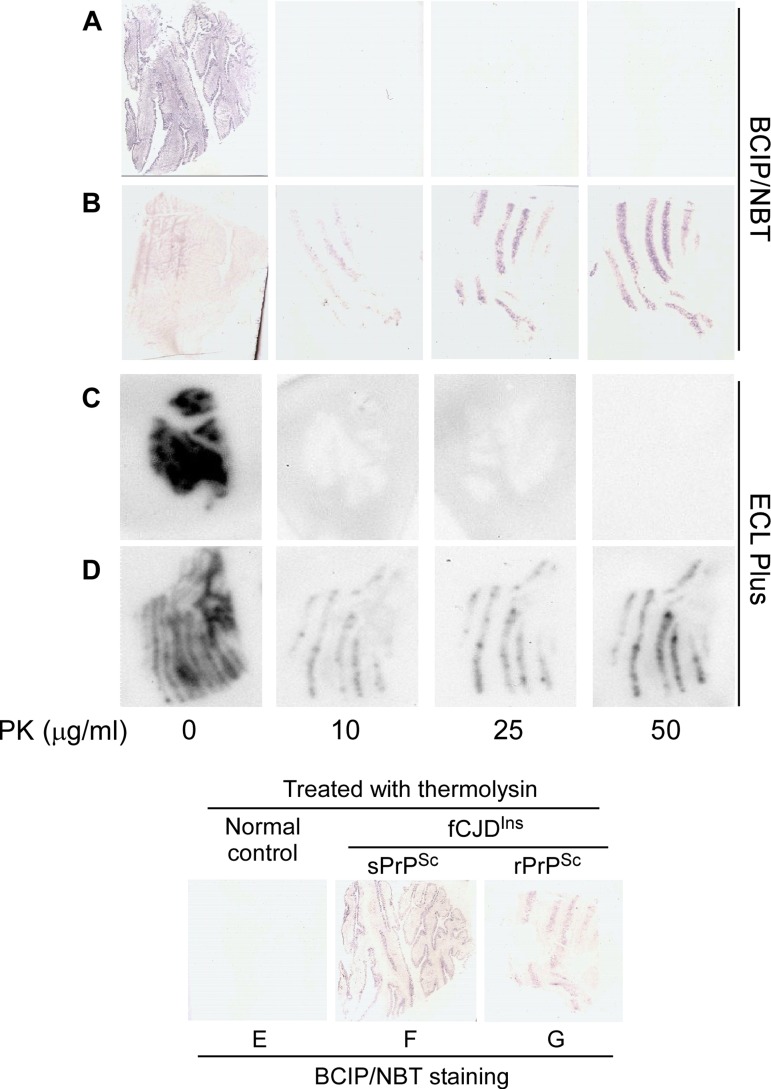
Histoblotting of fCJD^Ins+sPrPSc^ and fCJD^Ins+rPrPSc^. The nitrocellulose membranes blotted with tissue sections from the cerebellar cortex of fCJD^Ins+sPrPSc^ (**A**, **C**, and **F**) or fCJD^Ins+rPrPSc^ (**B**, **D**, and **G**) were developed with BCIP/NBT similar to classic IHC in **A, B, E, F, and G** while the membranes were developed with ECL Plus similar to Western blotting in **C** and **D**. PrP staining was detected in all blots without PK-treatment. After PK-treatment at either 10, 25, or 50 μg/ml, no PrP staining was detected in the samples from fCJD^Ins+sPrPSc^ (**A** and **C**), whereas PrP was detected in the samples from fCJD^Ins+rPrP^ (**B** and **D**). In contrast, PrP staining was detected in fCJD^Ins+sPrPSc^ (**F**) in addition to fCJD^Ins+rPrP^ (**G**) but not in normal controls after thermolysin that was reported to digest PrP^C^ only (**E**).

We investigated the PK-resistance of the PrP^Sc^ forming the stripes with two histoblot procedures: one based on IHC principles, the other similar to WB. Without PK-treatment, PrP was detected in the blots from cases with or without rPrP^Sc^ by both methods (Fig. 9, A-D). After PK-treatment with 10 or more μg/ml, PrP was detected only in cases with rPrP^Sc^ (Fig. 9B and 9D) but not in case 6 with sPrP^Sc^ (Fig. 9A and 9C). In contrast, after treatment with thermolysin, PrP was detected in all cases with fCJD^Ins^ regardless of the presence or absence of rPrP^Sc^ but not in normal controls (Fig. 9 E through G). Therefore, the histoblot results were consistent with the data from WB and with the conclusion that in case 6 the cerebellar stripes were made of sPrP^Sc^.

## DISCUSSION

Neurodegenerative disorders are all associated with misfolding of various cellular proteins [42-44]. Human prion diseases including sporadic, inherited and infectious forms are highly heterogeneous in terms of their broad range of clinical and pathological phenotypes [12]. In addition to the transmissible spongiform encephalopathy (TSE), non-transmissible prion diseases have also been reported [45-47], which has brought about a proposal that the spectrum of prion diseases should be beyond the classic definition of TSE [48]. The high heterogeneity of prion diseases may be associated with the chameleon-like conformations of the PrP^Sc^ molecule [11], the only component identified in the infectious prion pathogen to date [2]. Presence of a variety of rPrP^Sc^ including PrP^Sc^ type 1 and type 2 in sCJD and PrP7-8 in GSS detected by Western blotting and distinct brain PrP deposits detected by immunohistochemistry might be attributable to the variable PrP^Sc^ conformation [11, 12]. Recently, novel PK-resistant PrP species with a distinctive ladder-like gel profile have also been identified in a new prion disease termed VPSPr [34, 39]. These newly-identified PK-resistant PrP fragments are preferentially detected by 1E4 but much less immunoreactivity with the widely used 3F4, which is similar to those PK-resistant PrP species detected in the normal brain and uninfected cultured cells [33, 37, 49, 50]. Studies on the correlation between the phenotypic heterogeneity of the diseases and the chameleon-like conformation of PrP^Sc^ molecule are often complicated by the diversity in the etiologies of the diseases. Therefore, an investigation on cases with a single etiology will be critical for understanding the molecular mechanism responsible for this high heterogeneity in disease phenotypes.

Inherited CJD linked to the 144 bp insertion mutation provides us with an excellent opportunity to limit the influence of variable etiologies on phenotype. Since the disease is significantly and tightly linked to this single mutation [21, 22], the phenotypes of the disease should be mainly determined by the *de novo* generated-pathogen itself, mutation-containing PrP^Sc^. Compared to the observation by Mead et al. [24], our six cases had a similar mean age of death (44.0 ± 5.7 vs. 45.1 ± 7.3) while an elder mean age of onset (39.7 ± 8.8 vs. 34.9 ± 6.93). In our fCJD^Ins^ cases, five cases showed rPrP^Sc^ either similar to PrP^Sc^ type 1 or type 2 of sCJD. Strikingly, one case had no typical rPrP^Sc^ albeit the presence of clinical and phenotypic characteristics similar to other five cases. Instead, this case was demonstrated to be rich in sPrP^Sc^. The identification of the fCJD^Ins^ case rich in sPrP^Sc^ but lacking in typical rPrP^Sc^ raises several issues and implications as to molecular bases underlying the phenotypic heterogeneity and neuropathological changes of prion diseases.

### Effect of the levels and types of rPrP^Sc^ and sPrP^Sc^ on deposits of PrP patches and neuropathological changes

So far more than 100 individuals from at least eight families affected by the 144-bp insertion mutation have been identified around the world [21, 22, 51, 16, 24-29]. However, the reported examination of the brain PrP with Western blotting has been no more than nine cases in total to date, only six of which exhibited a detectable rPrP^Sc^ [24-26, 28]. The rPrP^Sc^ species examined with Western blotting were three heterozygotes with 129-M/V polymorphism and 144-bp insertion, in which two were similar to the Gambetti et al. sCJD PrP^Sc^ type 1 in both size and ratio of the three major PrP glycoforms [25] and one similar to PrP7-8 detected in GSS [28]. Although the two fCJD cases had an identical *PRNP*, one with a 4-year course exhibited only minimal focal spongiform degeneration and another with a 10-year course showed significant astrocytosis, neuronal loss and pronounced spongiform degeneration [25]. The levels of rPrP^Sc^ were five-fold less in the former than in the latter. Nevertheless, the deposits of PrP patches were equally detected in the molecular layer of the cerebellum of the two cases. In another study involving the largest known kindred so far with 86 affected individuals, PrP was examined by Western blotting in total five cases, of which a heterozygote with 129-M/V had the Collinge et al. PrP^Sc^ type 2 (corresponding to Gambetti et al. type 1 in terms of its gel mobility) and a homozygote with 129-M/M contained the Collinge et al. type 3 (corresponding to Gambetti et al. type 2) [24]. In addition, three out of five cases examined revealed no detectable rPrP^Sc^ by conventional Western blotting [24]. Interestingly, the heterozygote with Collinge et al. type 2 and a 19-year course (VI. 23 or their case 10) and the homozygote (129-M/M) with no rPrP^Sc^ and a 9-year course (VII.25 or their case 4) shared the same intense PrP patches in the cerebellum while the former exhibited slightly severer spongiosis and astrocytosis [23, 24]. Another case with144-bp insertion examined by Western blotting was reported by Gelpi et al. [26]. This case showed rPrP^Sc^ in a pattern resembling Gambetti et al. PrP^Sc^ type 1 although PrP^Sc^ type 2 might also be present. Neurohistologically, this case exhibited numerous eosinophilic globular structures in the molecular layer and the parahippocampal gyrus in addition to the spongiform changes, slight neuronal loss and gliosis as well as PrP diffuse synaptic staining [26]. The latest case examined by Vital et al was a heterozygote with 129-M/V polymorphism and was of GSS phenotype with PrP7-8 [28].

Our current study confirmed the marked variability in severity of neuropathological changes and levels of rPrP^Sc^ and the predictability in PrP patches staining, similar to previous observations [23-26, 28]. Nevertheless, our cases did not seem to reveal the close correlation either between the amount of rPrP^Sc^ and the length of disease duration or between the amount of rPrP^Sc^ and the severity of neuropathological changes. For instance, although the cases 2 and 3 had the longest and second longest disease durations (15- and 11-year, respectively) among the six cases we examined, the amounts of rPrP^Sc^ in the two cases were not the largest. The current characterization on six fCJD^Ins^ cases including two PrP^Sc^ type 2 and three PrP^Sc^ type 1 and one with no typical rPrP^Sc^ clearly demonstrated that the similar PrP patches are always equally detectable in fCJD^Ins^ regardless of the levels and types of rPrP^Sc^ except a case. It is conceivable that rPrP^Sc^ conformer should not be the main component of this type of PrP deposits and the abnormal sPrP^Sc^ conformer containing PrP^Ins^ participates in the formation of the PrP patches instead.

It is known that Western blot analysis is more sensitive than immunohistochemistry in terms of detection of a protein. However, for detection of the abnormal PrP, it has been reported in at least two conditions that immunohistochemistry readily detected the abnormal PrP staining in the samples in which the conventional Western blot analysis showed no detectable rPrP^Sc^ [34, 36]. For instance, by immunohistochemistry PrP deposition was readily detected in the olfactory mucosa of sCJD, where the amount of rPrP^Sc^ only accounted for as small as 8% of brain rPrP^Sc^ [36]. In these tissues, no rPrP^Sc^ was detected by conventional Western blotting. It is possible that the majority of PrP^Sc^ was composed of sPrP^Sc^ in the olfactory mucosa. Indeed, this was the case in VPSPr [34, 39]. Although there was strong PrP immunostaining with 3F4 in brains from the subjects with VPSPr, no rPrP^Sc^ was detected by the conventional Western blotting probed with the same antibody. The quantitative analysis revealed that the amount of rPrP^Sc^ was very small (as small as 10% of brain rPrP^Sc^ in sCJD) [34]. Therefore, it is most likely that PrP staining in the tissue section detected by immunohistochemistry is the signature of sPrP^Sc^ instead of rPrP^Sc^ alone. The pathognostic PrP patches in the fCJD^Ins^ may comprise sPrP^Sc^ deriving from both PrP^Ins^ and PrP^Wt^. Indeed, using histoblotting after PK- or thermolysin-treatment, we confirmed that staining of sPrP^Sc^ can be eliminated by PK but not by thermolysin. The latter is the enzyme that has been reported to specifically degrade PrP^C^ but not PrP^Sc^ including sPrP^Sc^ [52, 53]. Thus, our results suggest that sPrP^Sc^ may preferentially attack the cerebellum compared to the cerebrum.

### Allelic composition of total PrP and PrP^Sc^ in fCJD^Ins^

By using a PK-sensitivity assay, the rPrP^Sc^ molecule in fCJD with 144-bp insertion has been demonstrated to comprise both PrP^Ins^ and PrP^Wt^ alleles in a case with detectable rPrP^Sc^ [5]. However, whether both PrP^Wt^ and PrP^Ins^ participate in the formation of sPrP^Sc^ or not remains unknown. The ratios of PrP^Wt^ and PrP^Ins^ to the total PrP in fCJD^Ins^ are also unclear. We demonstrated that like rPrP^Sc^ in the typical fCJD^Ins+rPrPSc^, sPrP^Sc^ in fCJD^Ins+sPrPSc^ also derived from both PrP^Wt^ (~48%) and PrP^Ins^ (~52%). Although PrP^Wt^ and PrP^Ins^ accounted for 60% and 40% of total PrP, respectively, most of PrP^Ins^ (~62%) was converted into sPrP^Sc^ and 64% of PrP^Wt^ remained as PrP^C^. It is worth noting that less PrP^Wt^ became insoluble in the fCJD^Ins+sPrPSc^ case than in fCJD^Ins+rPrPSc^ (PrP^Wt^: ~45% vs. ~62%). Whether this is the reason that the insoluble PrP^Wt^ and PrP^Ins^ in the fCJD^Ins+sPrPSc^ did not form the typical rPrP^Sc^ remains to be confirmed. Compared to the previously reported case [5], the current study showed a greater percentage of insoluble PrP^Wt^ (~62% vs. ~57%) and smaller PrP^Ins^ (~70% vs. ~94%) in fCJD^Ins+rPrP^ [5]. The difference between the current study and the previous one may result from that we examined five cases here while only one case was examined in the previous study. Our 2D study further confirmed that PrP^Wt^ is predominant in fCJD^Ins+sPrPSc^. Whether PrP^Ins^ is less-expressed or it is readily degraded in fCJD^Ins^ remains to be determined. Surprisingly, both full-length PrP^Ins^ and PrP^Wt^ molecules share a similar *p*I at 9.0-9.5 and the difference between the two seems to be in the molecular weight only but not in the molecular charge. Another interesting finding is that although there are both PrP^Wt^ and PrP^Ins^ in fCJD^Ins^, the amount of PrP^Wt^ in fCJD^Ins^ is almost similar to that of PrP^Wt^ in non-CJD and sCJD.

### Pathophysiology of rPrP^Sc^ and sPrP^Sc^ species

The correlation between rPrP^Sc^ and the neuropathological changes is still controversial. The rPrP^Sc^ species detected in prion-infected brains are surprisingly not neurotoxic and PrP-knockout mice are resistant to prion infection [54, 55]. Moreover, subclinical forms of prion diseases have been observed in experimentally or naturally infected animals that harbor high levels of infectivity and PrP^Sc^ but are asymptomatic during a normal life-span [56, 57]. Conversely, wild-type mice inoculated with PrP^Sc^ of bovine spongiform encephalopathy showed no detectable rPrP^Sc^ in the brain despite the presence of neurological symptoms and neuronal death [58]. These conditions were observed not only in animals but also in humans. Fatal familial insomnia or GSS with substitution of valine for alanine at residue 117 (A117V) revealed striking clinical manifestations but little or undetectable PK-resistant PrP [59, 60]. Therefore, the molecular features of the neurotoxic forms of PrP remain to be determined. Several potentially toxic PrP isoforms have been studied in prion-infected transgenic mice, rodents and humans including transmembrane, cytosolic and PK-sensitive forms of abnormal PrP [61, 31, 62, 30]. Based on the “refolding” or “seeding” models, PrP^C^ may unfold to an intermediate before it refolds under the influence of PrP^Sc^ or the conversion of PrP^C^ into PrP^Sc^ requires a PrP^Sc^-like form (PrP*) [63, 64]. The intermediates have been widely observed in cell-based and cell-free models [65, 66, 41, 67]. These intermediates generated in the process of conversion of PrP^C^ to PrP^Sc^ could be the neurotoxic PrP species.

The PK-sensitive sPrP^Sc^ was initially proposed in experimentally infected animals using PTA-based ELISA by Safar et al. [31]. To our knowledge, our previous study was the first to demonstrate that human PrP^Sc^ is composed of both rPrP^Sc^ and sPrP^Sc^ and that the majority of PrP^Sc^ in GSS is sensitive to PK-digestion by using a PrP^Sc^-specific antibody-based Western blot analysis [30]. Currently the physiochemical features and pathophysiology of sPrP^Sc^ are poorly understood. The possibility cannot be ruled out that sPrP^Sc^ is an intermediate in the formation of the terminal product rPrP^Sc^ and it is responsible for the prion-related neurotoxicity. Remarkably, VPSPr-129VV that we recently identified is rich in sPrP^Sc^ and virally lacks the typical rPrP^Sc^ type 1 and type 2 and PrP7-8 in the cerebral cortex by conventional Western blot analysis [34, 39]. In the scrapie-infected hamsters, it has been shown that sPrP^Sc^ forms smaller oligomers while the rPrP^Sc^ forms the larger aggregates [62]. Our current finding that an fCJD^Ins^ with typical clinical and neuropathological characteristics is lack of typical rPrP^Sc^ but full of sPrP^Sc^ favors the hypothesis that the sPrP^Sc^ comprising small oligomers is most likely responsible for the neuropathological changes. Indeed, in Alzheimer's disease, the toxicity was originally thought to be a property of the fibrillar form of Aβ, consistent with the widespread notion at the time that the amyloid fibril itself was pathogenic [68, 69]. However, subsequent studies revealed that Aβ fractions containing protofibrillar comprising soluble oligomers, but not fibrillar, material retained their toxicity [70-72].

Like in non-CJD, uninfected cultured cells, and VPSPr, we detected rPrP in fCJD^Ins+sPrPSc^ as well when the 1E4 antibody-based Western blotting was used [33, 37, 34, 39, 50]. Although the gel profile of rPrP detected in non-CJD with 1E4 is similar to that of fCJD^Ins+sPrPSc^, the intensity of rPrP is much lower in non-CJD than in fCJD^Ins+sPrPSc^. The rPrP species in non-CJD was only visible in over-exposed films (data not shown). The gel profile of rPrP in fCJD^Ins+sPrPSc^ is different from that of VPSPr. Interestingly, it is more similar to that of rPrP^Sc^ in sCJD type 1at the lower PK concentrations ranging from 5 to 10 μg/ml with an unglycosylated PrP band migrating at ~19 kDa similar to sCJD type 2 while the typical gel profile of sCJD type 1 with an unglycosylated PrP band migrating at ~21 kDa becomes dominant at higher PK-concentrations greater than 25 μg/ml. It would be interesting to further investigate whether all 1E4-preferentially detected rPrP species originate from a similar precursor.

## MATERIALS AND METHODS

### Reagents and antibodies

Sodium phosphotungstic acid (NaPTA), proteinase K (PK), and phenylmethylsulfonyl fluoride (PMSF) were purchased from Sigma Chemical Co. (St. Louis, MO, USA). Thermolysin was purchased from Sigma. Peptide N-glycosidase F (PNGase F) was from New England Biolabs Inc. and used following manufacturer protocol. Urea, CHAPS, DL-dithiothreitol (DTT), Iodoacetamide (IAA), tributylphosphine (TBP), Ampholine pH 3-10, and immobilized pH gradient (IPG) strips (pH 3-10, 11 cm long) were from Bio-Rad (Richmond, CA, USA). Reagents for enhanced chemiluminescence (ECL Plus) were from Amersham Pharmacia Biotech, Inc. (Piscataway, NJ, USA). Magnetic beads (Dynabeads M-280, tosylactivated) were from Dynal Co. (Oslo, Norway). Anti-PrP antibodies, including rabbit anti-N-terminal antiserum against human PrP residues 23-40 (B. Ghetti, Indiana University, USA), anti-C-terminal antiserum immuno-reactive to human PrP residues 220-231 [59], and mouse monoclonal antibody 3F4 against human PrP residues 106-110 [73, 38], were used.

### Human brain tissues

Consent to use autopsy material for research purposes had been obtained for all samples. Autopsy was performed within 20 hours from the death. Clinical data and relevant hospital records were examined. Cases of proved non-CJD, sporadic CJD, and GSS diagnosed at the National Prion Disease Pathology Surveillance Center (NPDPSC, Cleveland, USA), were used as controls.

Brains, sent to the NPDPSC for suspected prion disease diagnosis, were obtained at autopsy and one half was immediately frozen and stores at −80 °C. The remaining tissue was fixed in formalin for 10 days, kept in 98 % formic acid for 1 h and again in formalin until sampling for neuropathological examination and PrP immuno-histochemistry [39]. The presence of PrP^Sc^ from frozen tissues of frontal (FC) and occipital (OC) cortices, brain stem (BS), and cerebellum (CE) were determined by western blotting. In addition, paraffin blocks of tissues from FC, OC, BS, and CE were prepared for histology and immunohistochemistry.

### Molecular genetics

The genomic DNA was extracted from frozen brain tissues. The open reading frame (ORF) of the *PRNP* was amplified by the polymerase chain reaction (PCR) using 20 ng of genomic DNA and primers PrPO-F (GTCATYATGGCGAACCTTGG, Y=C+T) and PrPO-R (CTCATCCCACKATCAGGAAG, K=T+G) (PCR cycles: 94°C for 3 min; 94°C for 1 min, 57°C for 1 min, 72°C for 1 min, 30 cycles; 72°C for 10 min). The PCR products were separated on a 1.0% agarose gel. Both the larger band (~0.9 kb) and the wild type size band (0.77 kb) were recovered separately from the gel using the QIAGEN gel extraction kit, and subjected to automated sequencing with primers PrPO-F, PrPO-R, and HP306R (CATGTTGGTTTTTGGCTTAC TC). For some samples where direct sequencing of PCR products did not give conclusive sequences, the PCR products were cloned then sequenced. The sequences were compared with that of the wild type human PrP using the LALIGN program (http://www2.igh.cnrs.fr/). The sequence of R3g is CCC CAT GGT GGT GGC TGG GGg CAG as defined by Goldfarb et al. [74].

### Preparation of brain homogenate, S2, and P2 fractions

The 10% (w/v) brain homogenates were prepared in 9 volumes of lysis buffer (10mM Tris, 150 mM NaCl, 0.5% Nonidet P-40, 0.5% deoxycholate, 5mM EDTA, pH 7.4) with pestle on ice. When required, brain homogenates were centrifuged at 1,000 g for 10 min at 4°C. In order to prepare S2 and P2 fractions, the supernatants (S1) were further centrifuged at 35,000 rpm (100,000 g) for 1 h at 4°C. After the ultracentrifugation, the detergent-soluble fraction was recovered in the supernatants (S2) while the detergent-insoluble fraction (P2) was recovered in the pellets that were resuspended in lysis buffer as described [33].

### Specific capture of PrP^C^ and PrP^Sc^ by 6H4 and g5p

The anti-PrP antibody 6H4 or DNA binding protein g5p (100 μg each) were conjugated to 7×10^8^ tosyl activated superparamagnetic beads (Dynabeads M-280, Dynal Co.) in 1 ml of phosphate-buffered saline (PBS) at 37 °C for 20 h, respectively. The conjugated beads were incubated with 0.1 % bovine serum albumin (BSA) in 0.2 M Tris-HCl at pH 8.5 to block non-specific binding. The prepared beads were stable for at least 3 months at 4°C. Brain homogenate (10%, w/v) was prepared in lysis buffer, followed by centrifugation at 3,000 g for 10 min at 4°C to remove debris. The specific capture of PrP^C^ or PrP^Sc^ by 6H4 or g5p was performed as described [41, 33] using brain homogenate and conjugated beads (10 μg mAb or g5p/6×10^7^ beads) in 1 ml of binding buffer (3% Tween-20, 3% Nonidet-40 in PBS, pH 7.4). After incubation with constant rotation for 3 h at room temperature, the beads were attracted to the sidewall of the plastic tubes by external magnetic force, allowing easy removal of all unbound materials in the solution. After three washes in wash buffer (2% Tween-20 and 2% Nonidet P-40 in PBS, pH 7.5), the beads were collected and were heated at 95°C for 5 min in SDS sample buffer (3% sodium dodecyl sulfate (SDS), 2 mM EDTA, 10% glycerol, 50 mM Tris-HCl, pH 6.8). The proteins eluted from the beads were subjected to SDS-PAGE and immunoblotting as described below.

### Precipitation of PrP^Sc^ by sodium phosphotungstate

Precipitation of PrP^Sc^ by sodium phosphotungstate (NaPTA) was conducted as described [32, 33] with mild modification. Briefly, 10% (w/v) brain homogenates from brain tissues were prepared in Dulbecco's sterile phosphate buffered saline (PBS) lacking Ca2+ and Mg2+. The gross cellular debris was removed by centrifugation at 1,000 rpm (80 g) for 1 min. Supernatant (500 μl) was mixed with an equal volume of 4% (w/v) sarkosyl prepared in PBS pH 7.4 and incubated for 10 min at 37°C with constant agitation. Samples were adjusted to final concentrations of 50 units/ml Benzonase (Benzon nuclease, purity 1; Merck) and 1 mM MgCl2 and incubated for 30 min at 37°C with constant agitation. Subsequently, the samples were adjusted with 81.3 μl of a stock solution containing 4% (w/v) NaPTA and 170 mM MgCl2 (prepared in water and titrated to pH 7.4 with sodium hydroxide) to give a final concentration in the sample of 0.3% (w/v) NaPTA. This stock solution was pre-warmed to 37°C before use. Samples were incubated at 37°C for 30 min with constant agitation before centrifugation at 14 000 rpm for 30 min. After careful isolation of the supernatant, the pellet was resuspended to 60 μl final volume of 1 X lysis buffer. The samples were incubated with PK at a final concentration of 50 μg/ml at 37°C for 1 h. Digestion was terminated by the addition of PMSF (3 mM final concentration) and boiling for 10 minutes in an equal volume of electrophoresis sample buffer (3% SDS, 2mM EDTA, 10% glycerol, 2.5% β-mercaptoethanol in 62.5 mM Tris, pH 6.8). After cooling for 2 min, the samples were incubated with a five-fold volume of pre-chilled methanol at −20°C for 2 h and centrifuged at 14,000 rpm for 20 min at 4°C. The supernatant was discarded and the pellet was resuspended in 30 μl sample buffer. The latter was subjected to SDS-PAGE and immunoblotting.

### Velocity sedimentation in sucrose step gradients

Brain homogenates (10%, w/v) in 1X Dulbecco's PBS pH 7.4 were mixed with an equal volume of 2X lysis buffer, then centrifuged for 10 min at 3,000 rpm at 4°C. Supernatants were collected and sarkosyl was added to 1% final concentration. Each sample was loaded atop of 10-60% step sucrose gradients and centrifuged at 200,000 × g in the SW55 rotor for 1 h at 4°C as described with minor modification [33]. After centrifugation, the content of the centrifuge tubes was sequentially removed from the top to the bottom to collect 12 fractions which were subjected to immunoblotting as described below.

### One- and two-dimensional gel electrophoresis and immunoblotting

Brain homogenates treated with or without PK were resolved either on 15% Tris-HCl Criterion (Bio-Rad) for one-dimensional (1D) PAGE or on pH gradient (IPG) strips for two-dimensional (2D) PAGE. The latter was performed as described by the supplier with minor modifications using the PROTEIN IEF cell (Bio-Rad) [33]. Briefly, for the 1D PAGE, samples boiled in 2X electrophoresis sample buffer were precipitated by 5-fold volume of pre-chilled methanol at −20 °C for 2 h, followed by centrifugation at 14,000 rpm for 20 min at 4°C. Pellets were resuspended in reducing buffer (8 M urea, 2% CHAPS, 5 mM TBP, and 20 mM Tris, pH 8.0) for 1 h at room temperature and then incubated with 20 mM IAA for 1 h. The samples were incubated with 5-fold volume of pre-chilled methanol at −20°C for 2 h and centrifuged at 14,000 rpm for 20 min at 4°C. Pellets were resuspended in 200 μl of rehydration buffer (7M urea, 2 M thiourea, 1% DTT, 1% CHAPS, 1% Triton X-100, 1% Ampholine pH 3-10, and trace amounts of bromophenol blue). Samples dissolved in rehydration buffer were incubated with IPG strips for 14 h at room temperature with shaking. The rehydrated strips were focused for about 40 kVh. For the second dimension, the focused IPG strips were equilibrated for 15 min in equilibration buffer 1 containing 6M urea, 2% SDS, 20% glycerol, 130 mM DTT, and 375 mM Tris pH 8.8, and then in equilibration buffer 2 containing 6M urea, 2%SDS, 20% glycerol, 135 mM iodoacetamide and 375 mM Tris pH 8.8 for another 15 min. The equilibrated strips were loaded onto 8-16% Tris-glycine Criterion gel (Bio-Rad).

The proteins on the gels were transferred to either Immobilon-P (PVDF, Millipore) or Immobilon-FL membranes (PVDF, LI-COR) for 2 h at 70V. For probing the PrP molecule, the membranes were incubated for 2 h at room temperature with 3F4 (1:40,000) or anti-C-terminal antibody (1:4,000) as primary antibody. Incubation with a secondary antibody was performed either with the horseradish peroxidase-conjugated goat anti-mouse antibody (1: 3,000) or IRDye 800CW conjugated goat anti-mouse (LI-COR). The PrP bands or PrP spots were visualized on either Kodak film by the ECL Plus as described by the manufacturer or Odyssey infrared imaging system (LI-COR^®^ Biosciences, NE, USA).

### PrP Immunohistochemistry and histoblotting

Tissue was fixed in formalin for 3 weeks. The following procedures were performed as described [33]. For histological preparations, brain sections were embedded in paraffin and stained with hematoxylin-eosin. For immunohistochemistry sections were deparaffinized, rehydrated, and immersed in 98% formic acid for 1 h at room temperature. Endogenous peroxidase was blocked by immersion in 8% hydrogen peroxide in methanol for 10 min. Sections were completely immersed in 1.5 mM hydrochloric acid and microwaved for 10 min. After rinsing, they were incubated with the mouse monoclonal antibody 3F4 at 1:600, washed and incubated with bridge antibody (goat anti-mouse, Cappel, 1:50) followed by incubation with mouse PAP complex (Sternberger, Meyer Immunocytochemicals, 1:250). Diaminobenzidine tetrahydrochloride was used to visualize the immunoreactivity.

The histoblot was prepared as previously described [75]. Four 8-mm-thick cryosections from each block were transferred to nitrocellulose membranes that had been dampened with lysis buffer (0.5% NP-40, 0.5% sodium deoxycholate, 100 mM NaCl, 10 mM EDTA, 100 mM Tris HCl pH 8.0). The membranes were thoroughly air dried, rehydrated for 30 min in 0.5% Tween 20 with phosphate-buffered saline (PBS-T), dampened with Guanidine hydrochloride 2M (in Millipore H_2_O) 25 min and handled as follows: 1) no proteases-treatment, 2) digestion with proteases: PK at 10, 25 and 50 μg/ml in lysis buffer (37°C for 1 h), or thermolysin 150 μg/ml (70°C, 1 h). The membranes were then treated in PBS-T containing 5% nonfat dry milk for blocking (30 min at room temperature). After being washed with PBS-T, membranes were incubated with the 3F4 monoclonal antibody (1:10,000) for 2 h at 37°C or overnight at 4°C. Binding was detected by incubation with either peroxidase-conjugated goat anti-mouse IgG antibody for 1 h at 37°C, and the reaction was developed with a chemiluminescence detection kit (ECL Plus, Amersham Biosciences, USA) or by phosphatase-conjugate goat anti-mouse IgG antibody for 1 h at 37°C, and the reaction was developed with BCIP/NBT(5-Bromo-4-chloro-3-indolyl hosphate/Nitro blue tetrazolium).
